# Mountaintop Removal Coal Mining Contaminates Snowpack across a Broad Region

**DOI:** 10.1021/acs.est.4c02596

**Published:** 2024-06-18

**Authors:** Colin A. Cooke, Kira M. Holland, Craig A. Emmerton, Paul E. Drevnick, Alison S. Criscitiello, Brandi Newton

**Affiliations:** † Environment and Protected Areas, Government of Alberta, 9888 Jasper Ave, Edmonton, Alberta T5J 5C6, Canada; ‡ Department of Earth and Atmospheric Sciences, University of Alberta, Edmonton, Alberta T6G 2E3, Canada; § Department of Biological Sciences, University of Alberta, Edmonton, Alberta T6G 2E9, Canada; ∥ Environment and Protected Areas, Government of Alberta, 3535 Research Road NW, Calgary, Alberta T2L 2K8, Canada; ⊥ Department of Biological Sciences, University of Calgary, Calgary, Alberta T2N 1N4, Canada

**Keywords:** polycyclic aromatic compounds, Elk River valley, air pollution, particulate matter, fugitive dust, selenium

## Abstract

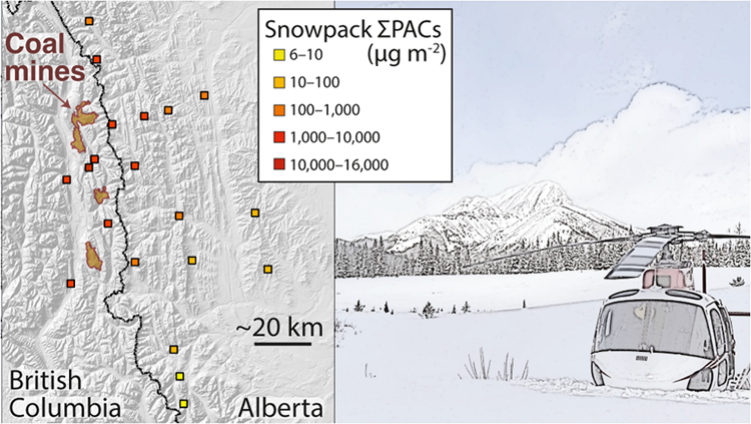

Mountaintop removal coal mining is a source of downstream pollution. Here, we show that mountaintop removal coal mining also pollutes ecosystems downwind. We sampled regional snowpack near the end of winter along a transect of sites located 3–60 km downwind of coal mining in the Elk River valley of British Columbia, Canada. Vast quantities of polycyclic aromatic compounds (PACs), a toxic class of organic contaminants, are emitted and transported atmospherically far from emission sources. Summed PAC (ΣPAC) snowpack concentrations ranged from 29–94,866 ng/L. Snowpack ΣPAC loads, which account for variable snowpack depth, ranged from <10 μg/m^2^ at sites >50 km southeast of the mines to >1000 μg/m^2^ at sites in the Elk River valley near mining operations, with one site >15,000 μg/m^2^. Outside of the Elk River valley, snowpack ΣPAC loads exhibited a clear spatial pattern decreasing away from the mines. The compositional fingerprint of this PAC pollution matches closely with Elk River valley coal. Beyond our study region, modeling results suggest a depositional footprint extending across western Canada and the northwestern United States. These findings carry important implications for receiving ecosystems and for communities located close to mountaintop removal coal mines exposed to air pollution elevated in PACs.

## Abstract 2

Snowpack samples from across the southern Canadian Rockies reveal widespread polycyclic aromatic compound pollution sourced from mountaintop removal coal mining.

## Introduction

Mountaintop removal coal mining in the Canadian Rocky Mountains is an important global source of metallurgical coal. In 2020, 80% of Canada’s annual metallurgical coal exports came from a series of four mines in the Elk River valley of British Columbia (BC), with the majority exported to the Asia-Pacific region.^1^ Metallurgical coal production has clear economic benefits for the mine operator and the provincial and federal governments. But mountaintop removal coal mining is also an iconic example of destructive resource extraction with negative environmental impacts. Entire watersheds are transformed by destroying mountain summits (or summit ridges) to expose and extract buried coal resources. Vast quantities of waste rock, which are piled in nearby valleys, are produced during the process. The subsequent leaching of heavy metals from waste rock pollutes downstream ecosystems.^2−6^ In the case of the Elk River valley, mine water runoff drains into the Elk River, which flows into Lake Koocanusa, a transboundary reservoir that extends from southern British Columbia into Montana (United States). Recent research has revealed increasing concentrations and annual loads of selenium, nitrate, and sulfate in the Elk River sourced from the coal mines.^5^ Nitrate concentrations in the Elk River have increased 784% since 1979, and the river now delivers ∼95% of Lake Koocanusa’s annual selenium budget.^5^

Mountaintop removal coal mining in the Elk River valley was also shown recently to release fugitive coal dust and associated contaminants to the atmosphere.^7,8^ These contaminants can be transported and deposited far from their emission sources. A sediment core from Window Mountain Lake, a small headwater lake located ∼10 km east of the Elk View Coal Mine, records a near-exponential increase in the deposition of polycyclic aromatic compounds (PACs) since ∼1970.^7^ This increase in PAC deposition to the lake paralleled the rise of coal production in the Elk River valley.^7^ Over this same period, the compositional profile of PACs in Window Mountain Lake sediment shifted from a pyrongenic (i.e., biomass burning) to a petrogenic (i.e., fossil fuel) signature that matched closely with coal samples from the Elk River valley. In addition to PACs, a recent doubling in the amount of selenium in the Window Mountain Lake sediment core was also noted. Petryshen^8^ subsequently reported measurements of selenium and other trace elements in moss tissues at 19 locations spread throughout the southern portion of the Elk River valley. Selenium concentrations in the moss were highest near the mines (PACs were not measured in the moss).

The two studies summarized above identified a previously undocumented vector for contaminants to be transported far from coal mine sources. However, the sediment core study only looked at one lake in a small (∼1 km^2^) watershed, while the moss study only assessed trace elements. Here, we use snowpack samples to examine spatial patterns in wintertime PAC and trace element deposition across a ∼ 10,000 km^2^ area spanning the North American Continental Divide. Snowpack integrates atmospheric deposition from the first snowfall to the time of sampling, with negligible postdepositional loss prior to spring nival melt. Focusing on winter deposition also eliminates significant input from wildfires, which can be an important source of both atmospheric particulate matter and pyrogenic PACs.^9^ Our results reveal, for the first time, clear evidence that coal mining contaminants are spread far downwind from their sources. Fugitive coal dust high in PACs is emitted from the Elk River valley coal mines and deposited at concentrations and at rates that rival some of Canada’s most impacted ecosystems.

## Methods

### Study Region and Snowpack Sampling

**Figure 1:**
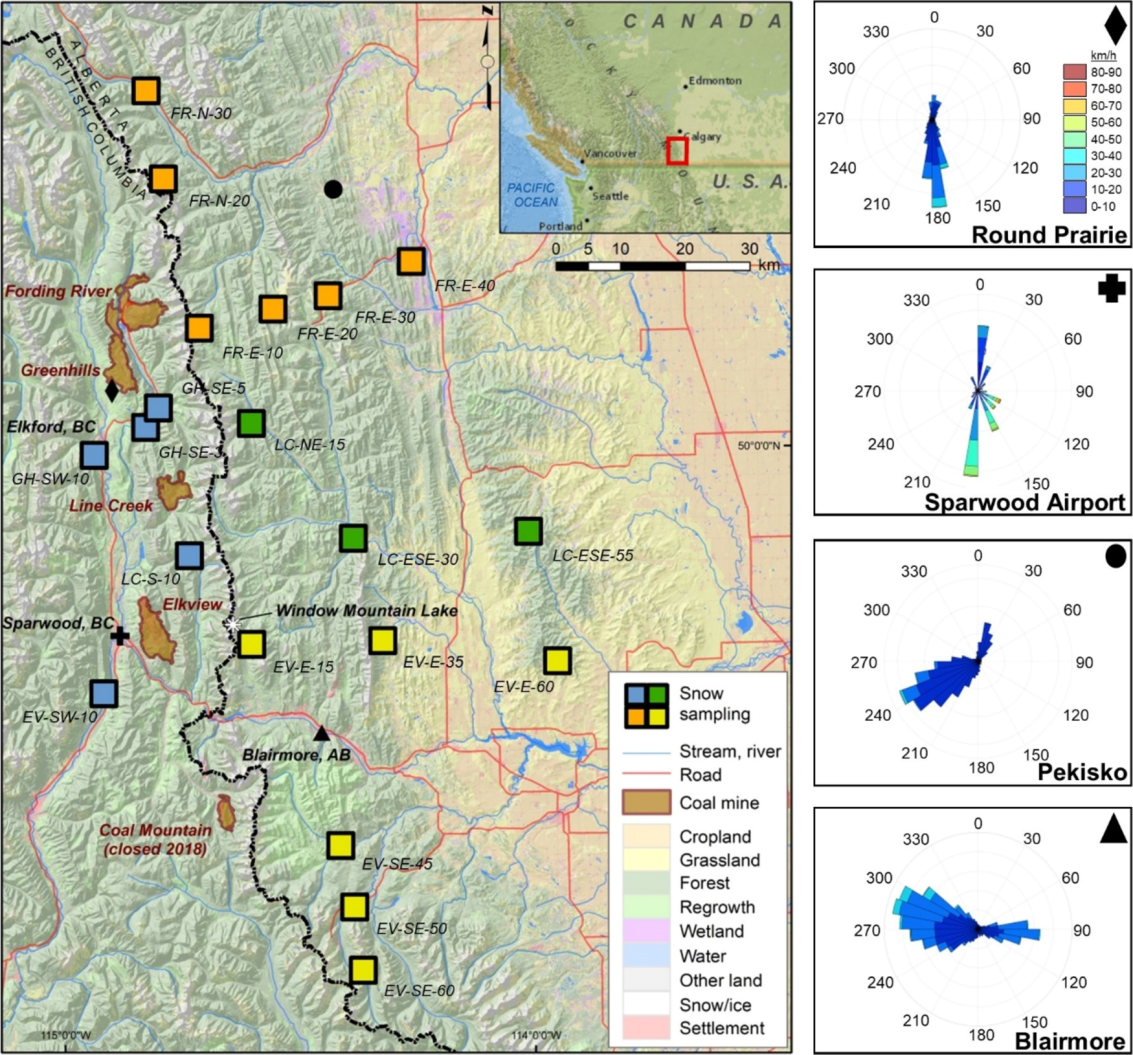
Map of the study region with snow sampling sites, the four Elk River valley coal mines, Window Mountain Lake, and regional wind monitoring locations. Snowpack sampling sites are grouped as either being within the Elk River valley (blue squares) or as part of transects moving away from the nearest mine. Wind rosettes show average wind direction and strength from November 1–May 31 (i.e., during the period of snow cover). The symbols in the wind rosettes correspond to each symbol’s location on the map.

The Elk River drains a 11,754 km^2^ valley that runs north–south along the western slopes of the Canadian Rocky Mountains (Figure 1). Four active mountaintop removal coal mines stretch along an ∼80 km north-to-south transect. The mines occur between ∼1500 and 2000 m elevation. Mountaintop removal coal mining in the Elk River valley began in 1970; coal production has increased steadily and now exceeds 20 million tons annually (Figure S1).^7,10^ Winds in this region are complex. Within the valley, wintertime winds blow mostly from the south; however, on the eastern side of the Continental Divide, winds are primarily from the west (Figure 1). These wind patterns facilitate the regional transport of atmospheric particles to the north and east.

To assess the history and magnitude of atmospheric pollution and deposition from mountaintop mining in BC, we collected snowpack samples from across the southern Canadian Rockies (Figure 1 and Table S1). Our sampling sites were located on both sides of the Continental Divide, which separates Pacific from Arctic and Atlantic drainages. In 2022, an initial seven snowpack samples were collected from four locations, with two of the four locations sampled in both February and March. In March 2023, 19 locations were sampled at varying elevations and distances from the coal mines. Snowpack sampling sites were roughly 10 km apart and along north-to-south and west-to-east transects away from the coal mines.

Snowpack in the region typically accumulates between early November and late March or early April (Figure S2). March is when snowpack typically reaches near-maximum depth prior to any spring melt. Sites were sampled from both within the Elk River valley and along a series of transects west to east away from the mines. Sites in the Elk River valley were sampled by road or by foot while sites in Alberta were accessed by helicopter. Daily sampling trips generally proceeded with more distant sites being sampled first and moving toward the mines. We attempted to sample at ∼10 km steps away from each mine but the mountainous terrain, weather conditions, and extensive tree cover imposed limitations on where we could safely fly and land. Samples were collected after engine shutdown and upwind of potential helicopter exhaust and downwash. Snowpack sampling followed established protocols.^11^ In brief, snowpits were dug to ground surface >50 m away from the landing location. Immediately after excavation, the shovel was cleaned in adjacent snowpack, and the snowpit was sampled from the snow surface continuously to the ground surface and placed into an 11 L bucket lined with a poly(tetrafluoroethylene) (PTFE, Teflon) bag, which was then ziptied closed and sealed with a bucket lid for transport. Care was taken to evenly sample the full depth of snowpack with no bias toward visibly dirty layers. Each sample therefore represents an integrated (mixed) sample of the entire snowpack. Sealed snow samples were kept at −36 °C until analysis.

Snowpack depth and density can be spatially heterogeneous, especially in mountainous regions.^12^ To account for this variability, snow water equivalent (SWE) was either obtained from automatic snow pillows deployed as part of the Alberta Snow Survey Network (Figure S2)^13^ or measured (*n* ≥ 3) at the time of sampling using a Federal snow sampler.^14^ Snowpack loads (in μg/m^2^) were calculated by multiplying contaminant concentrations (μg/L) by SWE (L/m^2^).

### Laboratory Analysis

Snowpack samples were returned frozen to the laboratory and melted at room temperature in the dark immediately prior to analysis. Samples for PACs were poured into airtight borosilicate jars and analyzed (unfiltered) at SGS AXYS Analytical Services (Sidney, BC, Canada) using EPA methods 1625C and 8270E within 14 days of melting.^15,16^ We measured concentrations of a broad suite of unsubstituted (parent) and alkylated PACs (Table S2), which can occur in high concentrations in coal.^7^ Summing the parent and alkylated PAC homologue concentrations yields a total PAC (ΣPAC) value. Individual PAC spike (surrogate) recoveries and field, procedural (i.e., pouring blank water over our sampling shovel), and laboratory blank results are presented in Tables S2 and S3. Field and shovel blank results indicate no PAC sorption to the shovel during sampling.

Selenium and other trace elements associated with particulate dust were also measured in 10 snowpack samples in both unfiltered water and after filtration at 0.45 μm. Unfiltered samples were preserved using 5% HNO_3_ and then microwave digested in closed vessels for 20 min at 180 °C before analysis by inductively coupled plasma mass spectrometry (ICPMS).^17^ Filtered samples were preserved following filtration and then analyzed by ICPMS. Laboratory protocols follow those employed in core provincial water quality monitoring in Alberta.^18^

### Air Mass Trajectory Analysis

Fugitive coal dust may be subject to long-range transport beyond the study region when entrained aloft. To evaluate the potential extent of coal dust transport, 1, 2, and 3 day forward trajectories were calculated for each of the four Elk River valley coal mines using the NOAA Hybrid Single-Particle Lagrangian Integrated Trajectory (HYSPLIT) model^19^ with North American Mesoscale Forecast System pressure-sigma hybrid level forecast data at 12 km resolution (NAM12).^20^ Trajectories were calculated for the snowpack period between October 2022 and April 2023. Trajectories were launched twice daily at half the height of the planetary boundary layer as calculated by HYSPLIT at times representative of both stable and convective boundary layer conditions. Trajectory end points, each representing 1 h of a trajectory, for all trajectories representing all four mines, were summed over an equal area grid to provide a composite residence time density map. While valley-scale meteorological conditions are likely not captured by the 12 km resolution of the NAM12 meteorological input data, we utilize HYSPLIT to identify general patterns of long-range transport over a large area. Valley-scale transport patterns are better captured by local wind data recorded at meteorological stations, as presented in Figure 1.

## Results

### Snowpack Contaminant Concentrations and Loads

**Figure 2:**
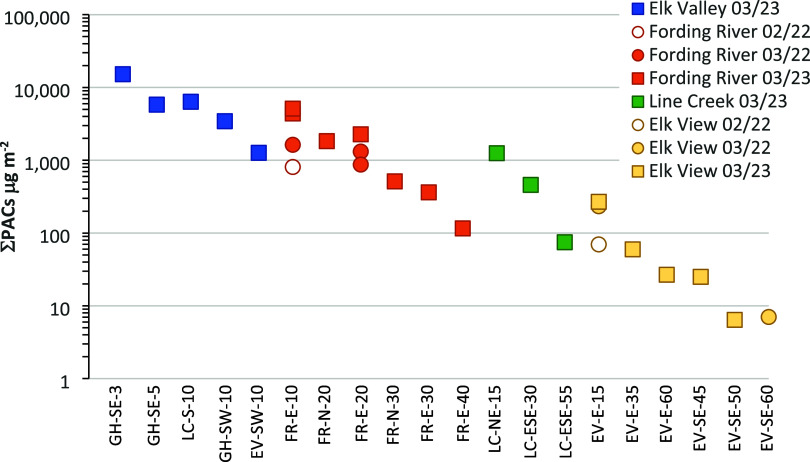
Snowpack summed polycyclic aromatic compound (ΣPAC) loads along the southern Canadian Rocky Mountains on a log scale. Sites are organized according to their location and the nearest Elk River valley coal mine with increasing distance from left to right. Snowpack was sampled in February and March 2022 and in March 2023. The first two letters of each site name refer to the nearest mine (GH = Greenhills; LC = Line Creek; EV = Elk View; FR = Fording River), followed by the general direction (e.g., SW = southwest), and by the approximate distance to the mine in km.

Snowpack ΣPAC concentrations ranged from 29 to 94,866 ng/L (Figure S3a), and snowpack loads ranged from 7 to 15,224 μg/m^2^ (Figure 2). Snowpack ΣPAC concentrations were above the various field, laboratory, and procedural blanks, which averaged 13 ng/L (range: 3–20 ng/L) (Table S3). Potential sources of error include intersite contamination and SWE estimations. Given the large volume of sample collected at each site (∼11 L of snow), the high PAC concentrations of the samples, and the low concentrations in our various blanks, potential contamination between sites appears negligible. Potential error in SWE estimations may affect PAC loading calculations. The snowpacks at all sites were dominated by faceted snow (i.e., depth hoar) with weak bonds, resulting in poor snowpack recovery with the Federal snow sampler due to snow loss through the side slots in the sampler, particularly if an ice lens was present in the snowpack. While care was taken to avoid this, the potential exists for underestimation of SWE; however, this would result in a conservative estimate of contaminant loadings.

Alkylated PACs were the dominant congeners, averaging 90% of ΣPAC concentrations (range: 80–92%) (Figure S3b). Four of our snowpack sampling sites were located at or near snow pillow monitoring locations, which provided hourly measurements of SWE (Figure S2). Samples from February and March of 2022 at two of these locations recorded increasing snowpack ΣPAC loads through the winter (Figure 2). For example, snowpack ΣPAC loads at EV-E-15 increased from 70 μg/m^2^ on February 2nd to 233 μg/m^2^ on March 29th (Figure 2). This suggests minimal loss of PACs from the snowpack during periods of snow accumulation and snowpack growth.

A clear spatial pattern was apparent in snowpack ΣPAC loads, with sites closer to the mines characterized by higher loads than those further from the mines (Figure 2). The highest ΣPAC loads were from within the Elk River valley. All five of our Elk River valley sampling sites had snowpack ΣPAC loads >1000 μg/m^2^, and site GH-SE-3 reached 15,224 μg/m^2^. This site is located <2 km from the Greenhills Mine coal processing facility, where coal is washed, dried, and loaded onto rail cars for transport to shipping terminals on Canada’s west coast (Figure S4). The very high ΣPAC deposition at this site likely reflects point-source emissions of coal dust lost during this processing. Snowpack ΣPAC loads at the four other Elk River valley sampling locations ranged from 1265 to 6358 μg/m^2^, with higher values found east of the Elk River. These results suggest intensive PAC deposition within the Elk River valley and especially along the east side of the river near the mines and the coal handling facilities.

Outside of the Elk River valley, our sampling transects provide insight into PAC transport and deposition away from each of the mines and over the Continental Divide (Figure 1). The highest snowpack ΣPAC loads were found ∼10 km east and north of the Fording River Mine (Figure 2). At FR-E-10, snowpack ΣPAC loads were 1631 μg/m^2^ in March 2022, while duplicate samples from March 2023 had snowpack ΣPAC loads of 4384 and 5114 μg/m^2^. Similar snowpack ΣPAC loads were noted another 10 km north (FR-N-20: 1832 μg/m^2^) and east (FR-E-20: 2271 μg/m^2^) of the Fording River Mine. These high values are roughly equivalent to snowpack loads within the Elk River valley itself (Figure 2). Further away from the Fording River mine, snowpack loads decreased to 362 μg/m^2^ at 30 km east and 116 μg/m^2^ at 40 km east.

A similar pattern is evident in transects east of the Line Creek and Elk View mines. Snowpack ΣPAC loads 15 km northeast of the Line Creek Mine (at LC-NE-15) were 1250 μg/m^2^, which is similar to snowpack ΣPAC loads at this distance east of the Fording River Mine (Figure 2). The same is true of snowpack loads at LC-ESE-30 (458 μg/m^2^) and LC-ESE-55 (75 μg/m^2^). Somewhat lower snowpack loads were noted in our transects away from the Elk View mine, which is the oldest and furthest south of the Elk River valley coal mines. Snowpack loads 15 km east of the Elk View Mine (EV-E-15) were 233 and 269 μg/m^2^ in March 2022 and 2023, respectively. These snowpack loads are ∼5× lower than those observed at a similar distance east of the Fording River and Line Creek mines. The lowest snowpack ΣPAC loads were observed to the 50–60 km southeast of the Elk View Mine at sites EV-SE-50 and EV-SE-60. Snowpack loads at these two sites were <10 μg/m^2^, and they had the lowest percent alkylated values (85 and 80%, respectively; Figure S3b).

The spatial patterns described above in snowpack ΣPAC loads likely reflect a mix of both predominant wind direction and emissions sources. Wintertime winds west of the Continental Divide blow predominately up-valley from the south, while winds east of the Continental Divide blow predominately from the west (Figure 1). These wind patterns result in emissions being transported north and east of the mines. In addition, the Fording River and Greenhills mines, which abut each other, accounted for roughly half of total Elk River valley coal production over the past three years (Figure S1b). This combination of wind patterns and high coal production in northern portion of the Elk River valley likely drive the spatial patterns we observe. They also suggest high PAC deposition is likely occurring in both Elk Lakes Provincial Park (in BC) and in Peter Lougheed Provincial Park (in Kananaskis Country in Alberta).

In contrast to PACs, snowpack samples were almost devoid of many trace elements, with most elements very close to or below the laboratory quantification limits (Figure S5). This includes selenium, with all results ≤0.2 μg/L in both filtered and unfiltered samples. Coal does contain trace elements, including many heavy metals that may be toxic at low concentrations.^21^ However, our results suggest that trace elements present in the coal dust are diluted by the high mountain snowfall amounts to levels that are below our method detection limits.

### Snowpack PAC Composition

**Figure 3:**
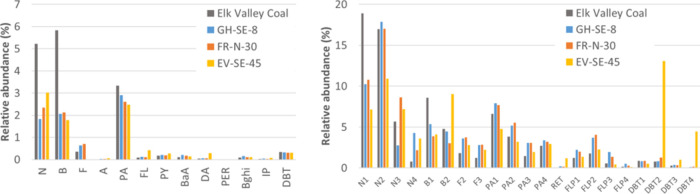
Relative abundance of various parent PACs (left panel) and alkylated PAC homologues (right panel) measured in a sample of Elk River valley coal and three representative snowpack samples. Most samples had a compositional profile that matched closely with Elk River valley coal, with the exception of a few snowpack samples collected east and south of the Elk View Mine (e.g., EV-SE-45). In these samples, additional sources of PACs with higher relative amounts of some alkylated species (e.g., C2-biphenyl and C2-dibenzothiophene) add to PAC inputs from fugitive coal dust. Note that not all individual congeners and homologues measured are shown. Abbreviations for the various PACs are defined in Table S2.

While PAC concentrations and loads varied over multiple orders of magnitude, PAC composition was remarkably similar across the various sampling sites (Figure 3). Moreover, the composition of snowpack PACs matched closely to the composition of Elk River valley coal, which we have reported on previously.^7^ Unsubstituted and alkylated homologues were dominated by naphthalene (N), biphenyl (B), phenanthrene (PA), and other lower-molecular-weight compounds. Despite the close agreement between coal and our snowpack samples, two important differences are evident. First, relative to the coal sample, the snowpack samples had lower concentrations of naphthalene. Loss of this low-molecular-weight compound may occur during coal mining, processing, fugitive dust transport, or postdeposition to the snowpack. Alternatively, our coal samples may not capture heterogeneity in PAC composition within the coal deposit. Second, snowpack samples collected east and south of the Elk View Mine (e.g., EV-SE-45) all contained higher relative concentrations of a few alkylated homologues, including C2-Biphenyl and C2- and C4-Dibenzothiophenes. These sampling locations are parallel to the Highway 3 transportation corridor, which runs through Crowsnest Pass. While a coal dust compositional profile is still clearly evident, these samples also appear to incorporate some amount of PAC pollution from other (petrogenic) sources. Overall, the snowpack PAC compositional profiles, along with the clear spatial patterns in PAC concentrations, reflect just how dominant the coal dust signature is in controlling the concentration and composition of PACs in snowpack across this broad region.

The results summarized above for snowpack ΣPAC concentrations, loads, and composition implicate the Elk River valley coal mines as the dominant source of wintertime PAC pollution across the southern Canadian Rocky Mountains. Fugitive coal dust is clearly being transported atmospherically over the Continental Divide and deposited far from the emission sources. At present, this PAC deposition footprint extends across the headwaters of the Elk River, the Oldman River, and into the headwaters of the Bow River.

Despite sampling far from the emission sources, nearly every snowpack sample we collected contained measurable PACs. The lowest snowpack ΣPAC concentrations and loads were noted in our two most distant sites (EV-SE-50 and EV-SE-60). Snowpack ΣPAC loads at these two sites were <10 μg/m^2^ (Figure 2), which may represent a regional background deposition rate. Snowpack ΣPAC loads 40 km east of the Fording River Mine and 55 km east of the Line Creek mine remained more than 10× this regional background value. This suggests our sampling failed to fully characterize the size of the impacted area.

**Figure 4:**
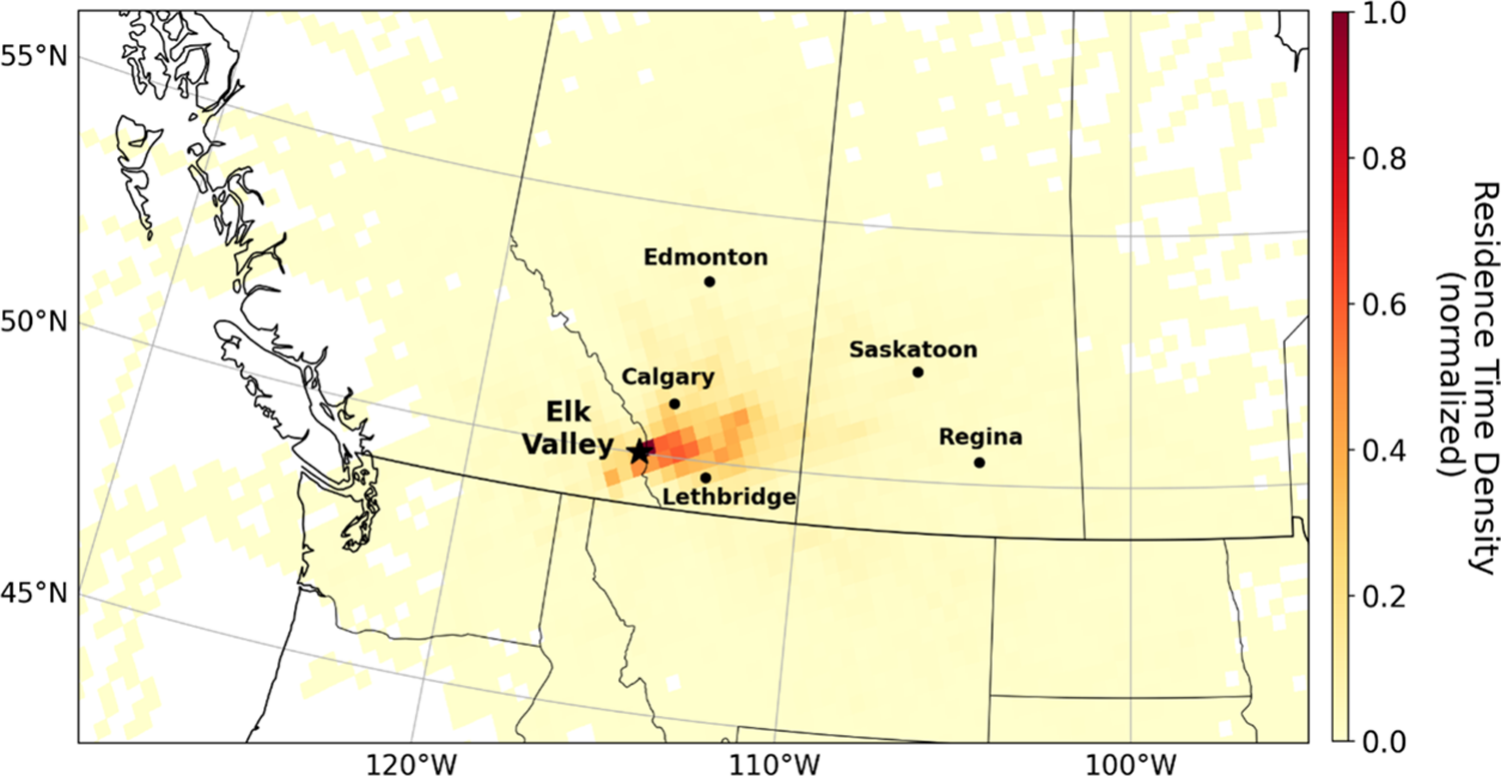
Modeled HYSPLIT forward air mass trajectories over 3 days at twice daily timesteps using North American Mesoscale Forecast System archived forecast data. The model results imply the potential for long-range transport of coal dust beyond our sampling region extending across western Canada and the northwestern United States.

Our HYSPLIT forward air mass trajectories suggest the potential for long-range transport of fugitive coal dust across western Canada and the northwestern United States (Figure 4). Winter air masses passing through the Elk River valley provide opportunities for fugitive coal dust transport away from the coal mines. Using the center point of each grid cell reveals that only one of 12 total grid cells with normalized residence time density values >0.4 lie within BC—the remainder are within Alberta (Figure S6). Elevated air mass residence time density values (>0.2 but <0.4) extend to the cities of Calgary and Lethbridge, raising the potential for population exposure far from the emission sources.

## Discussion

**Figure 5:**
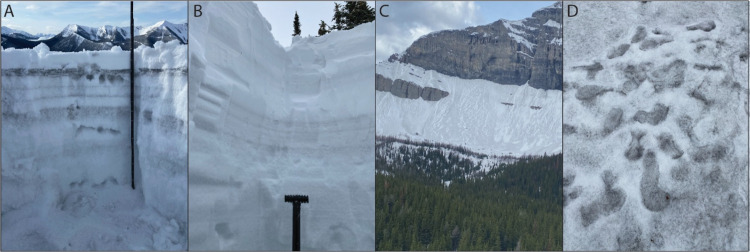
Photographs of late winter snowpits (A, B) and late spring snowpack (C, D). (A) Near site GH-SE-3 in the Elk River valley, where clear black coal dust depositional layers are present (photo: K. Holland, March 15, 2023). (B) At site FR-E-10, where high snowfall amounts dilute PAC deposition (photo: C. Cooke, March 14, 2023). (C) Looking toward FR-E-10 and (D) at FR-E-10 in spring (both photos: S. Campbell, May 31, 2022). Evidence for particulate coal dust is clearly evident darkening snow and becomes increasingly obvious as snowpack melts during the spring.

The snowpack contaminant data summarized above provide clear evidence that mountaintop removal coal mining in the Elk River valley is releasing vast quantities of PACs to the environment. The source of this PAC pollution is particulate coal dust. Coal dust is emitted to the atmosphere and transported far from emission sources before being deposited. Evidence for this mode of transport includes the clear spatial patterns described above with decreasing snowpack ΣPAC loads with distance from the mines. In addition, coal dust is visible as black horizons in the snowpack (Figure 5). In the Elk River valley, these black horizons are obvious in snowpack throughout the winter. Along Alberta’s eastern slopes, coal dust is most obvious in the spring when shrinking snowpacks accumulate and concentrate deposited material.

These results build upon similar sediment core PAC data from Window Mountain Lake, which is located east of the Elk View mine (Figure 1).^7^ A well-dated sediment core from Window Mountain Lake preserved a steady increase in PAC deposition that closely tracked 20th Century coal production in the Elk River valley. But lakes integrate material from both direct atmospheric deposition and watershed runoff, and delays in watershed export can result in accumulating legacy pollutant burdens in watershed soils.^22^ Snowpack samples, in contrast, represent an atmospheric signal uncomplicated by terrestrial inputs. Comparing lake sediment core and snowpack measurements of PAC deposition can therefore offer potential insight into watershed response and, specifically, the retention (or release) of PACs to aquatic ecosystems. In Window Mountain Lake, we noted a modern ΣPAC accumulation rate of ∼4500 μg/m^2^/y.^7^ At EV-E-15, which is located ∼3 km southeast of Window Mountain Lake (Figure 1), we observed a snowpack load of ∼250 μg/m^2^/y. Snowpack typically accumulates over a five-to-six-month period in this region (Figure S2). Assuming snow-free months are characterized by similar rates of PAC emissions and deposition, our snowpack data would suggest annual ΣPAC deposition of around 500 μg/m^2^/y to this region. This is roughly 10× lower than implied by the Window Mountain Lake sediment core. This suggests that either (i) summertime deposition vastly exceeds wintertime deposition, which seems unlikely, or (ii) watershed erosion of legacy PAC pollution—accumulated over decades of deposition—supplements contemporary atmospheric deposition to Window Mountain Lake. The implication is that at least some fraction of the PACs deposited are subsequently exported to downstream environments. While we cannot quantify the retention time of PACs deposited to the terrestrial environment, we fully anticipate the export of legacy PAC pollution, accumulated in watersheds over decades of coal mining, will continue long after coal mining and associated PAC emissions end.

The lack of detectable selenium in our snowpack samples stands in contrast to evidence for the emission and deposition of selenium (and other trace metals) in the Elk River valley. In a study of contemporary moss tissue from 19 locations surrounding the Elk View Mine, Petryshen^8^ found concentrations of selenium and 27 other elements varied as a function of proximity to the mountaintop mines. Similarly, we noted a small but steady increase in selenium concentrations and accumulation rates in the Window Mountain Lake sediment core, which, we speculated, was also due to fugitive coal dust deposition.^7^ However, the very low concentrations of selenium in Elk River valley mosses (all samples were <0.6 μg/g)^8^ and the lack of any detectable selenium in our snowpack samples (all samples were <0.2 μg/L; Figure S5) suggest atmospheric emissions of selenium and other trace metals are low and likely limited to highly localized deposition. Excess selenium supply is an important issue downstream of the Elk River valley coal mines.^5^ But, downwind of the mines, PACs are the primary contaminants of concern. This difference reflects the unique sources of these contaminants. High selenium in rivers and streams draining coal mines is sourced from the waste rock stockpiled on site. This waste rock contains sulfide minerals (pyrite, most commonly) and organosulfide compounds. Chemical weathering of this waste rock leads to the oxidation of the sulfide minerals and releases SO_4_^2–^ and associated trace elements, including selenium. In contrast, the PACs released into the atmosphere are primarily associated with the coal itself. While this may indicate differences in the downstream and downwind impacts of mountaintop removal coal mining, there is also clear potential for coal particles to be transported downstream (and especially during freshet). However, PACs are not currently part of aquatic ecosystem monitoring programs in the Elk River valley.^23^ Given high PAC loads in snowpack and the potential for watershed mobilization, we suggest downstream measurement of PACs—and especially alkylated PACs—is warranted.

Our study is the first to examine wintertime PAC pollution in a mountaintop removal coal mining region. But other studies have assessed snowpack PAC concentrations and loads in other remote and industrialized regions. Usenko et al.^11^ analyzed 17 PACs in late winter snowpack from across eight western United States National Parks. The highest snowpack loads were noted in Glacier National Park, which is immediately south of our study region. They observed snowpack loads of 310 μg/m^2^ at Snyder Lake (west of the Continental Divide) and 40 μg/m^2^ at Oldman Lake (east of the Continental Divide). Usenko et al. suggested this PAC air pollution was sourced from an aluminum smelter located southwest of Glacier National Park. Summing the same 17 PACs in our snowpack samples yields values 0.5 and 1.5 μg/m^2^ in our two most southern sampling sites (EV-SE-45 and EV-SE-60) (Figure S7). These values are much lower than those observed by Usenko et al. in Glacier National Park.^11^ However, 16 of the 17 PACs measured by Usenko et al. are parent (unsubstituted) PACs, with the only exception being Retene, a C4-Phenanthrene sourced primarily from biomass burning. The PACs measured by Usenko et al. only account for between 4 and 9% of the PACs quantified in our snowpack samples (Figure 3). Thus, by not measuring a broader suite of alkylated PACs, Usenko et al. may have missed an important source of PACs to Glacier National Park and the northwestern United States.

Outside of the mountains, snowpack sampling for PACs has been an important part of monitoring around the Alberta oil sands open-pit mines.^24−28^ Snowpack samples collected as part of oil sands monitoring have been analyzed at the same analytical laboratory using identical methodologies to our snowpack samples, allowing for a direct comparison. Between 2011 and 2017, snowpack ΣPAC loads in the oil sands region declined with distance from the open-pit mines, averaging 4020 μg/m^2^ at sites <10 km from the mines, 536 μg/m^2^ at sites 10–30 km from the mines, and 147 μg/m^2^ at sites 30–50 km from the mines.^26,27^ These values are quantitatively similar to our results for the southern Canadian Rockies. Our results reveal PAC pollution around the Elk River valley coal mines at a magnitude and spatial extent that rivals deposition around the Alberta oil sands.

Our results underscore recent efforts to broaden and standardize the quantification of PAC species—and particularly alkylated species—considered in environmental assessments.^29^ Alkylated species and especially heterocyclic PACs (i.e., those containing an oxygen, nitrogen, or sulfur atom within a ring) have proven useful for source characterization.^26,30^ Future studies in this region (and of environmental matrices, broadly) should consider analyzing for a broader suite of PACs as additional compounds offer opportunities for improved source characterization.

### Implications for Air Quality and Mining Communities

Regional air monitoring requirements imposed by the Government of British Columbia and the Government of Canada do not require the majority mine owner and operator, Teck Resources Limited, to report on atmospheric emissions of PACs. However, emissions of total particulate matter have been reported to Canada’s National Pollutant Release Inventory (NPRI).^31^ Between 2006 and 2021, annual particulate matter emissions increased >10-fold, rising from 11,618 to 164,339 tons. In 2021, fugitive dust accounted for 83% of total particulate matter emissions with road dust accounting for the remainder.^31^

Exposure to atmospheric particulate matter is the leading environmental threat to human health,^32^ and a growing body of evidence demonstrates varying toxicity of particulate matter emitted from different sources.^33^ The Government of British Columbia sets ambient air quality objectives for particulate matter.^34^ Regional air monitoring by Teck Resources Ltd. shows that ambient air quality generally meets these objectives.^35^ However, Teck’s stations are generally located upwind of the mines, with no stations located north or east of the Fording River Mine where we find deposition to be the highest. Teck is also not required to measure or characterize PACs as part of their annual air quality monitoring program. Thus, the ambient air quality objectives and regional monitoring program are not protecting areas downwind from poor air quality and dust deposition, nor are they protecting sensitive receptors from PACs in the coal dust.

The deposition of fugitive coal dust will likely continue for decades with cascading ecosystem consequences. At current production rates, coal reserves at each of the four operating Elk River valley coal mines are estimated to last another 15 years at the Line Creek Mine to 47 years at the Greenhills Mine.^10^ Assuming this mining proceeds, which is likely given multiple applications for expanding activities,^36^ we would anticipate an increase in the extent and magnitude of PAC pollution. Fugitive coal dust also contributes to a darkening of regional snowpack (Figure 5). This darkening will accelerate and exacerbate snowpack loss,^37^ which carries important implications for a region already stressed by declining winter duration and changing precipitation patterns.^38^ Some of this fugitive coal dust is undoubtedly sequestered in terrestrial soils and vegetation; however, our comparison with the Window Mountain Lake sediment core record suggests at least some of this material is exported to downstream environments. Snowmelt (freshet) seems an obvious period when this influx might occur, given other evidence for rapid solute^39^ and black carbon^40^ transport and downstream water quality response.

Our study focused on snowpack as a receptor of contaminant inputs to a montane ecosystem. But this region also hosts communities, the residents of which are exposed to this fugitive coal dust daily. Previous research in coal mining communities in the United States and elsewhere has documented increased rates of lung cancer mortality,^41^ cardiovascular disease,^42^ frequency of birth defects,^43^ and reduced quality of life.^44^ Importantly, these studies accounted for other known drivers of health outcomes, including smoking, poverty, education, age, sex, race, and other covariates. We know of only one study to assess mountaintop removal coal mining community exposure to atmospheric PACs explicitly. Hendryx et al.^45^ used silicone wristbands and passive polyurethane foam (PUF) samplers to assess personal exposure to atmospheric PACs. The authors demonstrated that mining community residents were exposed to elevated atmospheric PACs compared to nonmining communities. However, Hendryx et al.^45^ only examined nine (parent) PACs; our results suggest future assessments should incorporate a broad suite of both parent and alkylated compounds. To our understanding, no such studies have been conducted on communities in the Elk River valley or Crowsnest Pass. When compounded with well-documented water quality impacts in the Elk River valley, our results underscore a need to better understand the link between water and air pollution and human health across this region.

## Supporting Information Available

Total coal production (Figure S1); daily snow water equivalent (Figure S2); summed polycyclic aromatic compound (ΣPAC) concentrations (Figure S3); a photograph of the Greenhills coal loading facility (Figure S4); snowpack trace element concentrations (Figure S5); modeled HYSPLIT forward air mass trajectories (Figure S6); and the sum of the same 17 PACs (Figure S7); snowpack sampling sites and measured snow water equivalents (Table S1); a list of the various unsubstituted (parent) and alkylated PAC homologues (Table S2); and snowpack ΣPAC concentrations in the various field, laboratory, and procedural blanks (Table S3) (PDF)
