# Supplementary material for: Mountaintop Removal Coal Mining Contaminates Snowpack across a Broad Region
Source: Environ Sci Technol. 2024 Jun 18;58(26):11718–26. doi: 10.1021/acs.est.4c02596 (PMC11223467; doi:10.1021/acs.est.4c02596)
Supplement: Supplementary file 1 — es4c02596_si_001.pdf [file es4c02596_si_001.pdf]

Supporting Information

Mountaintop removal coal mining contaminates snowpack across a broad region

Colin A. Cooke[1,2,*], Kira M. Holland[2], Craig A. Emmerton[1,3], Paul E. Drevnick[4,5], Alison S. Criscitiello[2], Brandi Newton[4]

[1]Environment and Protected Areas, Government of Alberta, 9888 Jasper Ave, Edmonton, Alberta, T5J 5C6, Canada

[2]Department of Earth and Atmospheric Sciences, University of Alberta, Edmonton, Alberta T6G 2E3, Canada

[3]Department of Biological Sciences, University of Alberta, Edmonton, Alberta T6G 2E9, Canada

[4]Environment and Protected Areas, Government of Alberta, 3535 Research Road NW, Calgary, Alberta, T2L 2K8, Canada

[5]Department of Biological Sciences, University of Calgary, Calgary, Alberta T2N 1N4, Canada

*Corresponding author: colin.cooke@gov.ab.ca

12 pages, 7 figures and 3 tables.

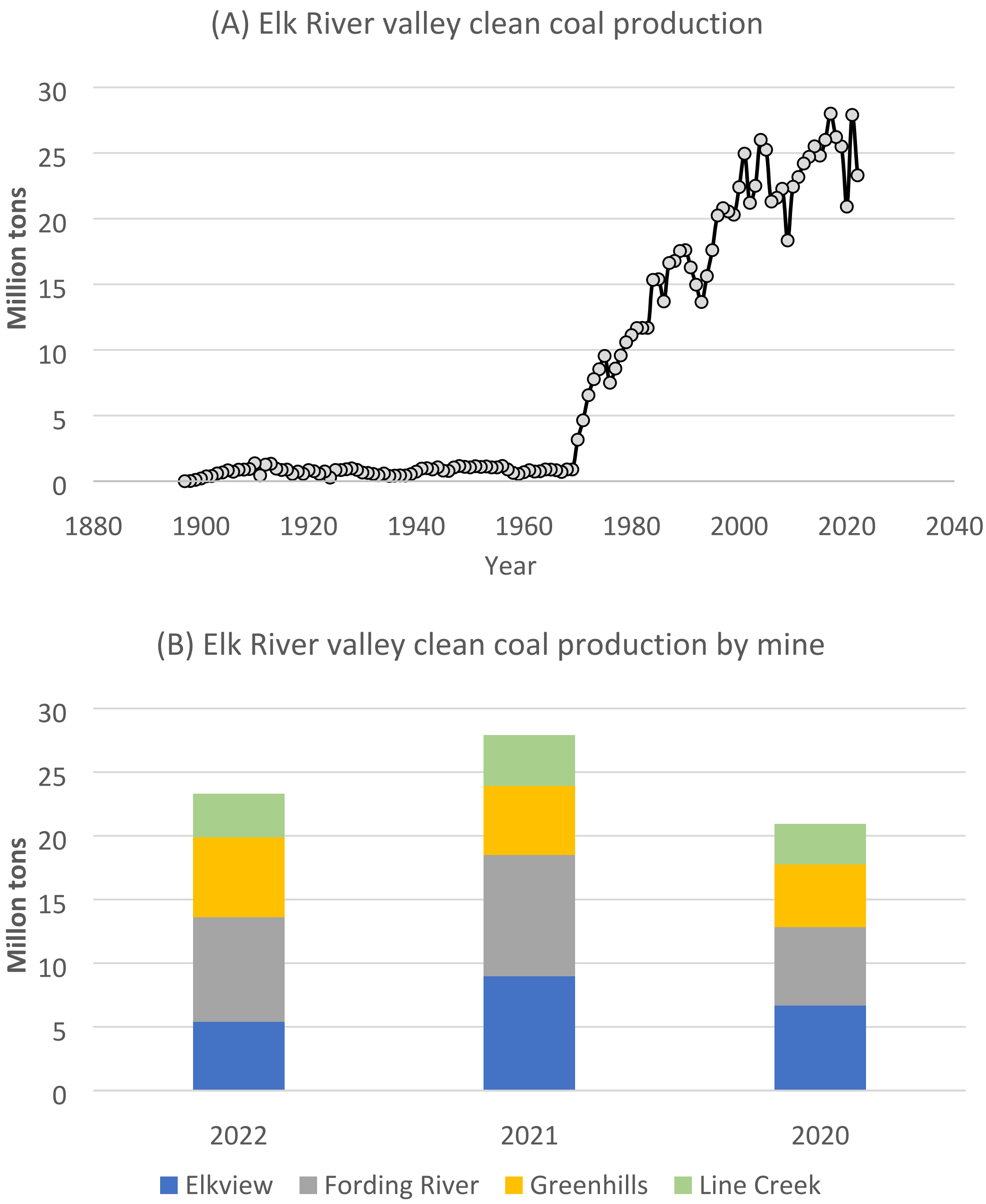

Figure S1. Total coal production through time in the Elk River valley (A) and over the past three years at the four Elk River valley coal mines (B) in million tons of clean coal.[1] The rapid increase in coal production in the 1970s reflects the onset of mountaintop removal coal mining. Together, the Fording River and Greenhills mines have accounted for roughly half of total coal production over the past three years.

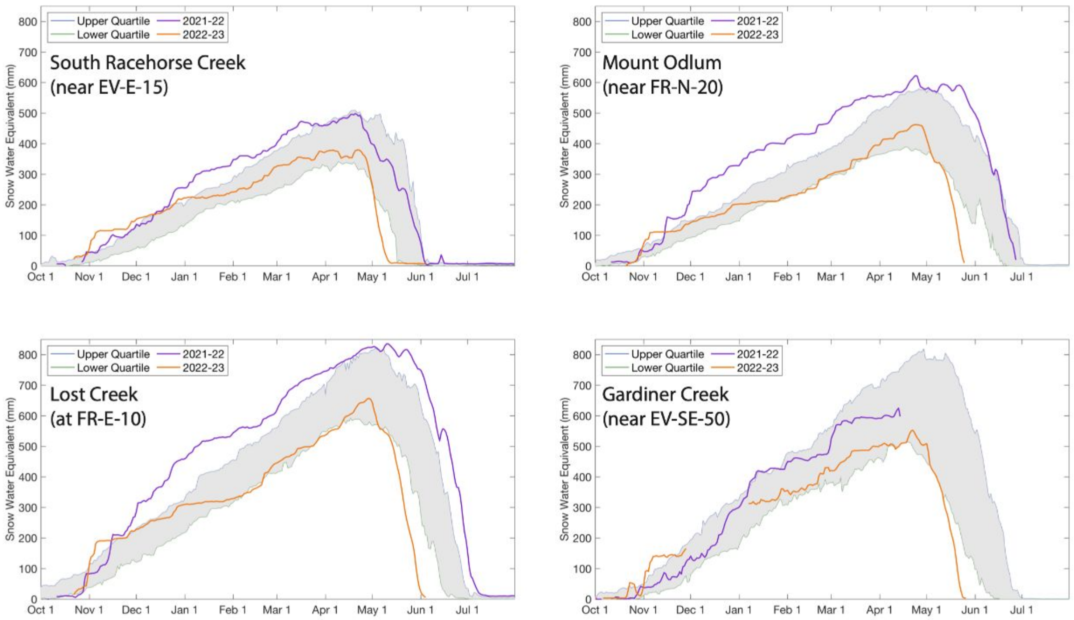

Figure S2. Daily snow water equivalent (SWE) for 2021–2022 and 2022–2023 along with the range of historical quartiles for each station. Snow pillow SWE data are from: https://rivers.alberta.ca/.

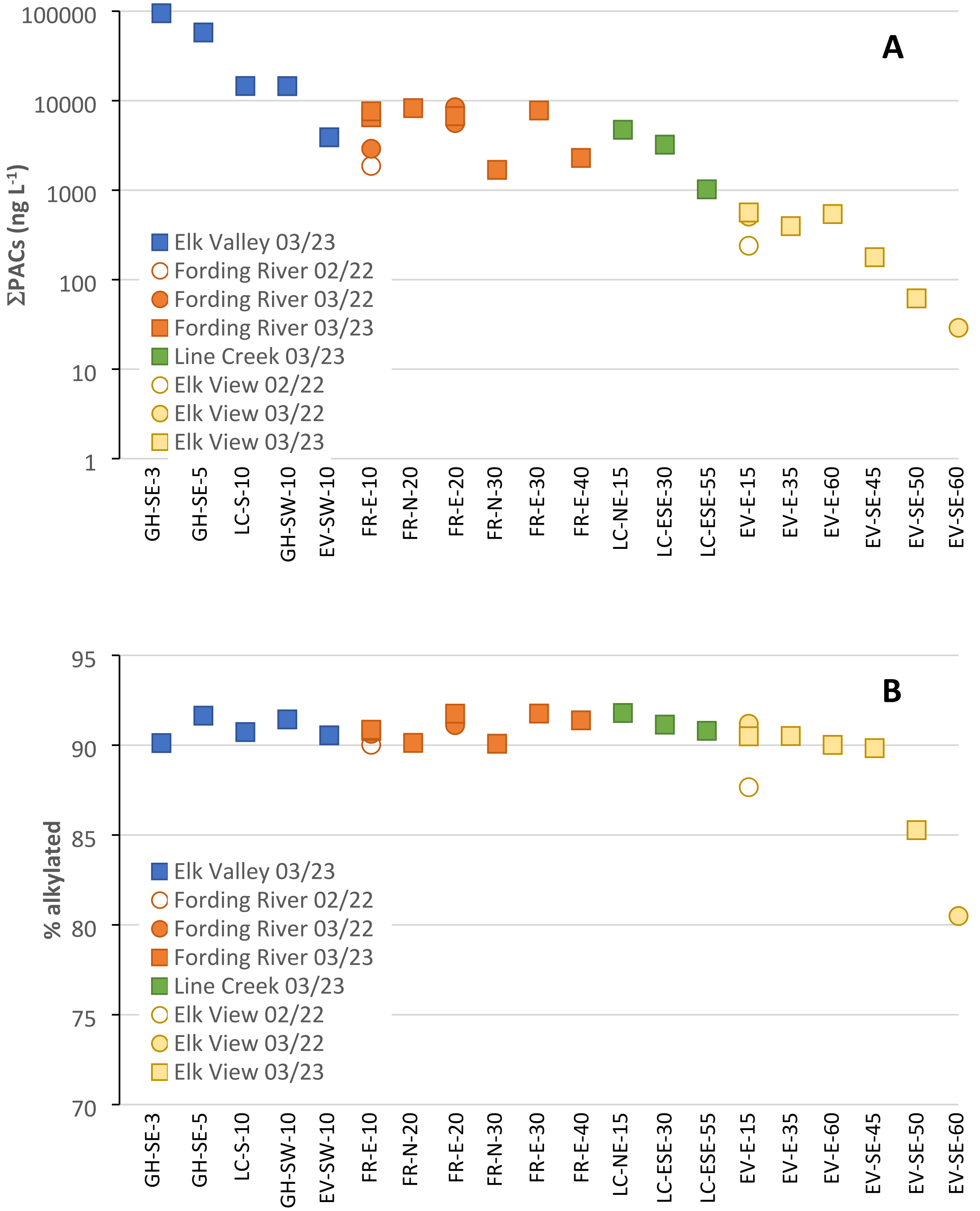

Figure S3. (A) Summed polycyclic aromatic compound (ΣPAC) concentrations for all 2022 and 2023 snowpack samples on a log scale. (B) The percent alkylated of the ΣPAC concentration on a linear scale.

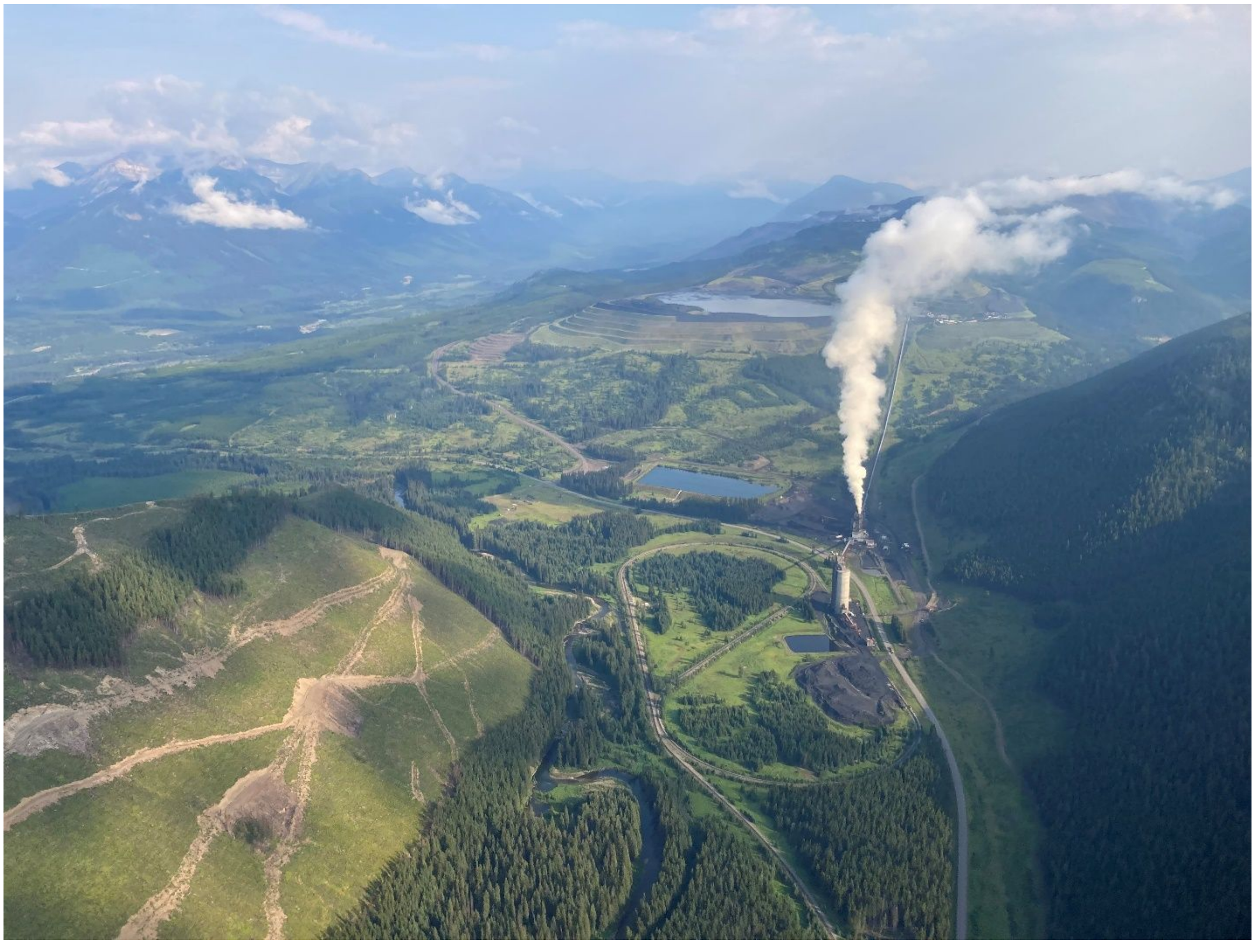

Figure S4. Photograph of the Greenhills coal loading facility (during summer) looking north up the Elk Valley.

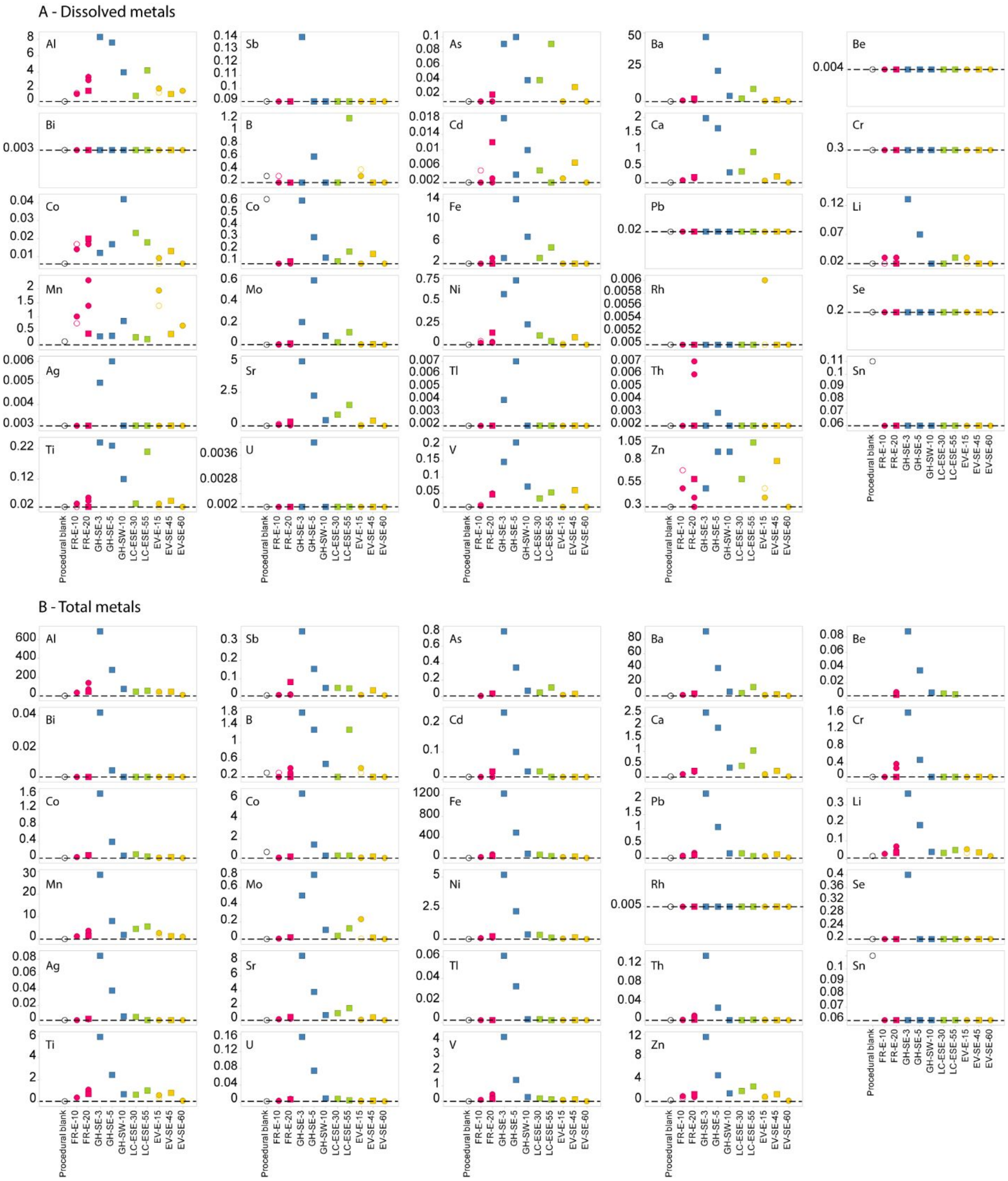

Figure S5. Snowpack trace element concentrations in both (A) dissolved (filtered) and (B) total (unfiltered) fractions in µg/L**.** Not all snowpack samples were analyzed for trace elements. Also shown is the reporting limit (RL, also known as the method detection limit) and concentrations in the procedural blank. Many elements were below the RL in virtually every sample analyzed (e.g., total and dissolved Se, dissolved Pb, etc.)

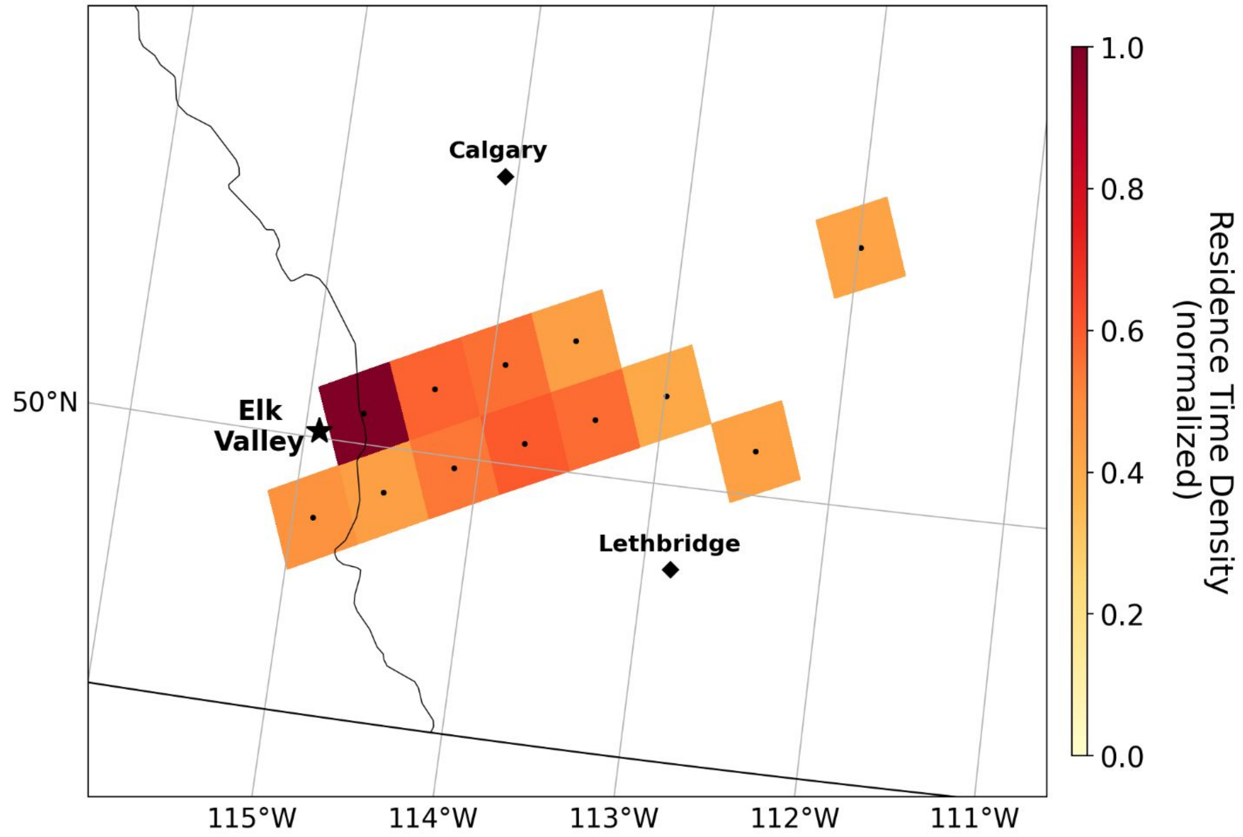

Figure S6. Modeled HYSPLIT forward air mass trajectories over three days at twice daily timesteps using North American Mesoscale Forecast System archived forecast data. In contrast with Figure 4, in this projection only grid cells with residence time density (normalized) values >0.4 are shown. Using the center of each grid cell (black dots) reveals that one of twelve grid cells lie within BC – the remainder are located within Alberta.

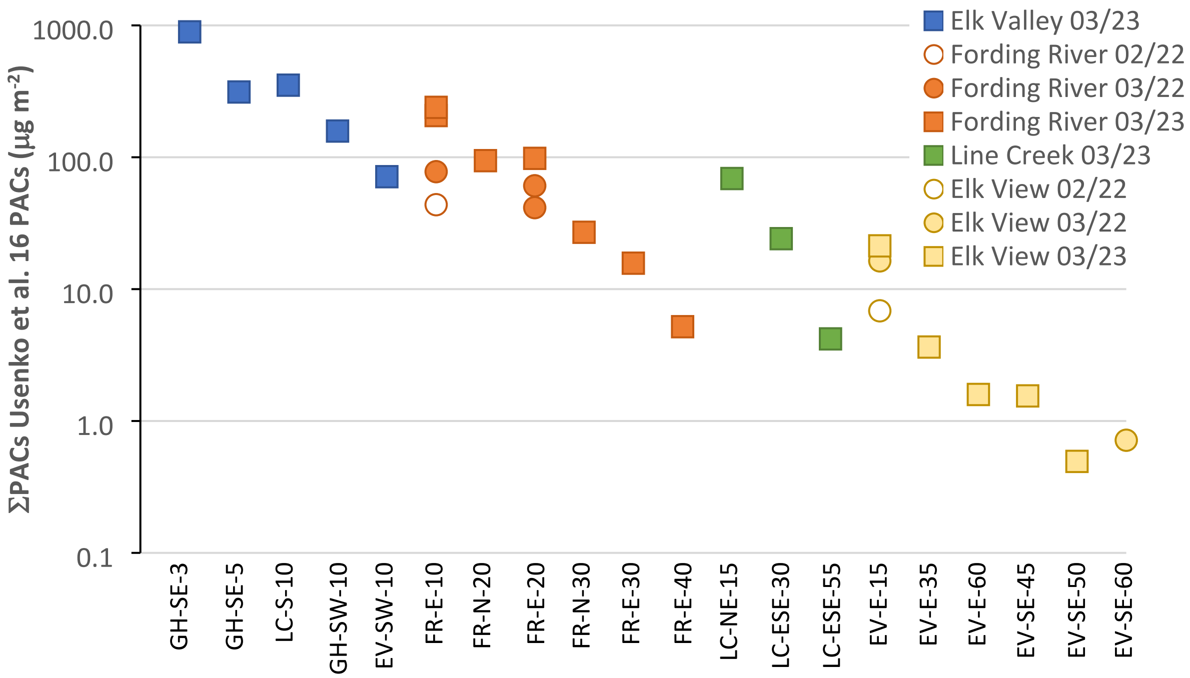

Figure S7. The sum of the same 17 PACs measured by Usenko et al.[2] in their study of western United States National Parks. Usenko et al. reported ΣPAC loads of 310 µg/m^2 at Snyder Lake (west of the Continental Divide) and 40 µg/m^2 at Oldman Lake (east of the Continental Divide). However, Usenko et al. measured 16 parent (unsubstituted) PACs plus retene. They did not measure alkylated homologs, which are the dominant species in our samples and in coal (see Figure 3 in the main text).

Table S1. Snowpack sampling sites and measured snow water equivalents (SWE). Site names list the nearest mine (GH = Greenhills; EV = Elk Valley; FR = Fording River; LC = Line Creek), the cardinal direction to the sampling site from the mine, and the distance to the nearest mine in km. For example, site GH-SE-8 is located approximately 8 km southeast of the Greenhills Mine. Sites marked with an * indicate a duplicate snow pit dug at the same general sampling location.

Name	Latitude	Longitude	Sampling date (m/d/y)	Snowpit depth (cm)	SWE (mm)
GH-SE-3	50.036	-114.827	3/15/2023	85	160
GH-SE-5	50.063	-114.799	3/15/2023	37	100
LC-S-10	49.857	-114.733	3/15/2023	75	435
EV-SW-10	49.665	-114.918	3/15/2023	65	324
EV-SW-10	49.997	-114.938	3/15/2023	47	236
FR-E-10	50.174	-114.710	2/2/2022	190	432
FR-E-10	50.174	-114.710	3/29/2022	193	560
FR-E-20	50.200	-114.550	3/29/2022	47	155
FR-E-20*	50.200	-114.550	3/29/2022	47	155
FR-E-10	50.174	-114.710	3/14/2023	36	670
FR-E-10*	50.174	-114.710	3/14/2023	40	670
FR-N-20	50.382	-114.787	3/14/2023	45	221
FR-E-20	50.200	-114.550	3/13/2023	39	337
FR-N-30	50.505	-114.824	3/14/2023	26	303
FR-E-30	50.218	-114.430	3/13/2023	14	46
FR-E-40	50.265	-114.249	3/14/2023	12	50
LC-NE-15	50.041	-114.598	3/14/2023	28	263
LC-ESE-30	49.880	-114.380	3/13/2023	39	142
LC-ESE-55	49.887	-114.002	3/13/2023	20	73
EV-E-15	49.733	-114.600	2/2/2022	100	291
EV-E-15	49.733	-114.600	3/29/2022	140	459
EV-SE-60	49.278	-114.366	3/29/2022	115	242
EV-E-15	49.733	-114.600	3/13/2023	70	476
EV-E-35	49.737	-114.318	3/14/2023	26	150
EV-SE-45	49.452	-114.412	3/13/2023	40	140
EV-E-60	49.706	-113.943	3/14/2023	11	49
EV-SE-50	49.366	-114.383	3/13/2023	30	104

Table S2. List of the various unsubstituted (parent) and alkylated PAC homologs measured as part of this study and average percent recovery of spike (surrogates).

Parameter	Parameter Short Name	Grouping	Mean (±1SD) % Recovery
Naphthalene	N	parent	-
Biphenyl	B	parent	-
Acenaphthylene	AY	parent	-
Acenaphthene	AE	parent	-
Fluorene	F	parent	-
Anthracene	A	parent	-
Phenanthrene	PA	parent	-
Fluoranthene	FL	parent	-
Pyrene	PY	parent	-
Benz[a]anthracene	BaA	parent	-
Chrysene	C	parent	-
Benzo[b]fluoranthene	BbF	parent	-
Benzo[j,k]fluoranthenes	BjkF	parent	-
Benzo[e]pyrene	BePY	parent	-
Benzo[a]pyrene	BePY	parent	-
Dibenz[a,h]anthracene	DA	parent	-
Perylene	PER	parent	-
Benzo[ghi]perylene	BghiP	parent	-
Indeno[1,2,3-cd]pyrene	IP	parent	-
C1-Naphthalenes	N1	alkyl	-
C2-Naphthalenes	N2	alkyl	-
C3-Naphthalenes	N3	alkyl	-
C4-Naphthalenes	N4	alkyl	-
C1-Biphenyls	B1	alkyl	-
C2-Biphenyls	B2	alkyl	-
C1-Acenaphthenes	AE1	alkyl	-
C1-Fluorenes	F1	alkyl	-
C2-Fluorenes	F2	alkyl	-
C3-Fluorenes	F3	alkyl	-
C1 Phenanthrenes/Anthracenes	PA1	alkyl	-
C2 Phenanthrenes/Anthracenes	PA2	alkyl	-
C3-Phenanthrenes/Anthracenes	PA3	alkyl	-
C4-Phenanthrenes/Anthracenes	PA4	alkyl	-
C1-Fluoranthenes/Pyrenes	FLPY1	alkyl	-
C2-Fluoranthenes/Pyrenes	FLPY2	alkyl	-
C3-Fluoranthenes/Pyrenes	FLPY3	alkyl	-
C4-Fluoranthenes/Pyrenes	FLPY4	alkyl	-

C1-Benzo[a]anthracenes/Chrysenes	BaAC1	alkyl	-
C2-Benzo[a]anthracenes/Chrysenes	BaAC2	alkyl	-
C3-Benzo[a]anthracenes/Chrysenes	BaAC3	alkyl	-
C4-Benzo[a]anthracenes/Chrysenes	BaAC4	alkyl	-
C1-Benzofluoranthenes/Benzopyrenes	BFP1	alkyl	-
C2-Benzofluoranthenes/Benzopyrenes	BFP2	alkyl	-
Dibenzothiophene	DBT	DBT	-
C1-Dibenzothiophenes	DBT1	DBT	-
C2-Dibenzothiophenes	DBT2	DBT	-
C3-Dibenzothiophenes	DBT3	DBT	-
C4-Dibenzothiophenes	DBT4	DBT	-
Naphthalene-D8	-	-	18(±10)
2-Methylnaphthalene-D10	-	-	22(±9)
Biphenyl-D10	-	-	27(±9)
2,6-Dimethylnaphthalene-D12	-	-	28(±9)
Acenaphthylene-D8	-	-	32(±8)
Dibenzothiophene-D8	-	-	36(±12)
Phenanthrene-D10	-	-	51(±7)
Fluoranthene D10	-	-	68(±6)
Benz[a]Anthracene-D12	-	-	77(±6)
Chrysene-D12	-	-	74(±6)
Benzo[b]Fluoranthene-D12	-	-	76(±8)
Benzo[k]Fluoranthene-D12	-	-	72(±7)
Benzo[a]Pyrene-D12	-	-	72(±7)
Perylene-D12	-	-	73(±7)
Dibenzo[a,h]Anthracene-D14	-	-	68(±12)
Indeno[1,2,3-C,D]Pyrene-D12	-	-	69(±9)
Benzo[g,h,i]Perylene-D12	-	-	68(±9)

Table S3. Snowpack ΣPAC concentrations in the various field, laboratory, and procedural blanks collected as part of this study. The procedural blank included pouring laboratory blank water over our sampling shovel and into our sampling container.

Sample	Date (m/d/y)	ΣPAC_{parent} (ng/L)	ΣPAC_{alkyl} (ng/L)	ΣPAHs (ng/L)
Lab Blank	2/2/2022	1.8	1.4	3.2
Lab Blank	3/29/2022	3.3	7.9	11.2
Procedural blank	2/2/2022	3.9	6.4	10.3
Field Blank 2023	3/13/2023	5.3	14.6	19.9
Lab Blank 2023	3/13/2023	3.7	14.3	18.0

References

(1) Cooke, C. A.; Drevnick, P. E. Transboundary Atmospheric Pollution from Mountaintop Coal Mining. *Environ. Sci. Technol. Lett.* **2022**, *9* (11), 943–948.

(2) Usenko, S.; Simonich, S. L. M.; Hageman, K. J.; Schrlau, J. E.; Geiser, L.; Campbell, D. H.; Appleby, P. G.; Landers, D. H. Sources and Deposition of Polycyclic Aromatic Hydrocarbons to Western U.S. National Parks. *Environ. Sci. Technol.* **2010**, *44*, 4512–4518.
